# Case Report: Longitudinal analysis of local immunoregulatory mediators in a DLBCL vitreoretinal lymphoma receiving local chemotherapy

**DOI:** 10.3389/fmed.2026.1786294

**Published:** 2026-06-10

**Authors:** Maria Carmela Saturno, Danilo Iannetta, Alessandro Lambiase, Marc D. de Smet

**Affiliations:** 1 Department of Sense Organs, Sapienza University of Rome, Rome, Italy; 2 Retina Associates, MIOS SA, Lausanne, Switzerland; 3 Icahn School of Medicine, New York Eye and Ear Infirmary of Mount Sinai, New York, NY, United States

**Keywords:** B-cells, biomarkers, cytokines, intravitreal methotrexate, lymphoma, vitreoretinal lymphoma

## Abstract

**Background:**

Vitreoretinal lymphoma (VRL) is a rare, high-grade B-cell lymphoma that affects immune-privileged sites and is classified as a variant of primary central nervous system lymphoma (PCNSL). The pathogenesis of VRL, including the origin of malignant B-cells, remains poorly understood. Cytokines, chemokines, and growth factors are thought to play critical roles in both the homing and autocrine proliferation of lymphomatous B-cells. In this study, we present the first longitudinal analysis of the dynamic interplay of vitreous immune mediators during intravitreal (IVT) methotrexate and dexamethasone therapy in a patient with VRL.

**Methods:**

A patient in their 70s with unilateral VRL underwent nine serial vitreous samplings over 12 weeks of IVT therapy with methotrexate (460 μg/injection) and dexamethasone (0.4 mg/injection). Each 0.2 mL vitreous sample was analyzed using a multiplex panel assessing 58 cytokines, chemokines, and growth factors. Vitreous levels were monitored longitudinally and correlated with clinical responses and optical coherence tomography.

**Results:**

During therapy, key cytokines, including IL-10, IL-6, IL-16, IL-1RA, and sIL-2R, decreased progressively, with IL-10 becoming undetectable by day 22. Chemokines CXCL12 and CXCL13, involved in malignant B-cell homing, declined more slowly than IL-10, suggesting persistent microenvironmental support for tumor cells. MCP-1 remained elevated, whereas hepatocyte growth factor (HGF) was consistently high, indicating potentially aggressive behavior. Other immune mediators, including MIP-1α, MIP-1β, Mig (CXCL9), and IP-10 (CXCL10), showed variable kinetics, reflecting a dynamic interplay between the tumor and host immune response. Notably, certain cytokines previously reported in VRL cohorts, such as FGF2, IFN-γ, TNF-α, and IL-17, were not elevated, highlighting patient-specific variability in the immunological profile.

**Conclusion:**

This case represents the first longitudinal characterization of vitreous immune mediators during IVT MTX/dexamethasone therapy for VRL. Dynamic changes in soluble mediators mirrored clinical tumor regression and suggested potential biomarkers for disease activity, therapeutic response, and prognosis. Persistent chemokines, such as CXCL12 and CXCL13, may indicate the optimal treatment duration, whereas high HGF levels may identify patients with aggressive disease. These findings propose a novel framework for integrating intraocular immunomonitoring into personalized therapeutic strategies and deepen our understanding of the complex tumor–immune microenvironment in vitreoretinal lymphoma. As derived from a single case, these observations are hypothesis-generating and require confirmation in larger cohorts.

## Introduction

Vitreoretinal lymphoma (VRL) is a rare, high-grade B-cell lymphoma of immune-privileged sites. In 2022, the World Health Organization, under its HAEM5 classification, recognized VRL as a variant of primary central nervous system lymphomas (PCNSLs). VRL predominantly belongs to the activated B-cell (ABC) subtype of diffuse large B-cell lymphoma (DLBCL) and is characterized by a high rate of somatic hypermutation (SHM) of the rearranged immunoglobulin heavy chain (IGH) gene, frequent coactivation of Toll-like receptor (TLR) and B-cell receptor (BCR), and constitutive activation of the NF-κB pathway (1, 2).

The source of malignant cells in primary VRL (PVRL) remains unclear, as does the molecular mechanism by which malignant B-cells home to immune-privileged sites, such as the eye and brain, where resident B-cells are typically absent (3). In a mouse model, chemokines facilitate migration to the subretinal space and subsequent cell proliferation (4). Notably, interleukin-10 (IL-10) serves as both an autocrine growth factor for tumor proliferation and survival and as a key immune escape mechanism (5).

Current treatment regimens for localized ocular involvement include intravitreal (IVT) methotrexate (MTX) or Rituximab, alone or in combination, with the possible addition of dexamethasone. For bilateral or central nervous system (CNS) involvement, local injections are adjunctive to systemic chemotherapy. Following an induction phase of weekly injections, the frequency may be adjusted based on the clinical response or residual IL-10 levels in the aqueous or vitreous humor (1). Response markers are essential for guiding the frequency and duration of therapy. In PCNSL, chemotherapy fails in 15%–25% of patients, with relapse in 25%–50% (3), and PVRL is similarly difficult to manage (1). A meta-analysis reported progression-free survival rates of 83% at 1 year and 58% at 2 years (6).

Cytokines, chemokines, and growth factors play crucial roles in the homing and autocrine proliferation of CNS/ocular lymphomatous B-cells (7). CXCL9, CXCL12, and CXCL13, by their perivascular expression, appear to hold particular significance in allowing homing for primary CNS/VRL lymphomas and tumor-infiltrating lymphocytes (8). CXCL13 in the cerebrospinal fluid (CSF) has diagnostic, therapeutic, and prognostic relevance in PCNSL (9).

Valuable insights into the immunoregulatory environment can be gained by monitoring patients after chemotherapy (10).

If one were able to serially sample CSF or vitreous humor during chemotherapy, it would be possible to determine the relative contribution of the host versus the neoplasm in the perpetuation of a deviant immune system. Although the CNS is not easily accessed or sampled, serial sampling in a previously vitrectomized eye can be safely performed during intravitreal chemotherapy.

We report a case of a patient with VRL in which longitudinal monitoring using a multiplex cytokine panel was performed during IVT MTX/dexamethasone therapy.

## Case description

**FIGURE 1:**
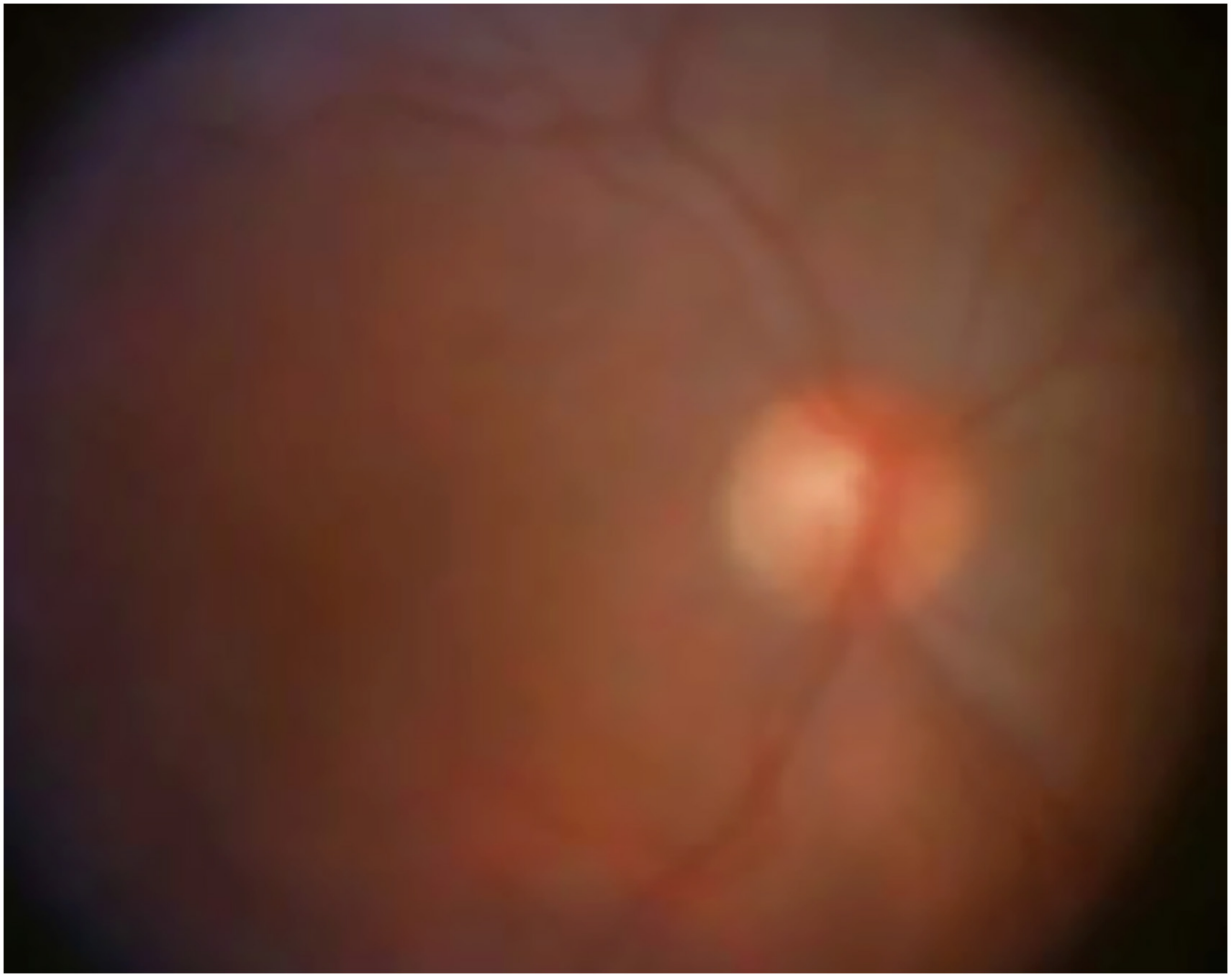
Fundus photograph showing dense vitreous haze obscuring retinal visualization at initial presentation.

A patient in their 70s was referred for unilateral decrease in visual acuity over 3 months. Dense vitreous haze precluded the retinal visualization (Figure 1). During the initial vitrectomy, multifocal cream-colored retinal spots were observed. Unfortunately, the sample was not sent for analysis, as the referring ophthalmologist diagnosed persistent vitreous hemorrhage. Subsequent optical coherence tomography (OCT) revealed subretinal hyperreflective infiltrates, and fluorescein angiography revealed hypofluorescent spots with window defects (“leopard skin pattern”). Contrast-enhanced brain magnetic resonance imaging revealed small supratentorial T2-hyperintense white matter lesions, initially thought to be vascular.

From a follow-up vitrectomy a month later, analysis of undiluted vitreous sample revealed an elevated IL-10/interleukin-6 (IL-6) ratio > 10 and positive MYD88. Cytopathology confirmed the diagnosis of B-cell lymphoma. IVT therapy with MTX (100 μg/mL vitreous volume; 460 μg per injection) and dexamethasone phosphate (0.1 mL of 4 mg/mL) was administered at regular intervals (Table 1) for 12 weeks with clearing of the subretinal and intravitreal tumor masses. At week 12, a CNS lesion was noted, and systemic chemotherapy was initiated. The patient decided to discontinue ocular follow-up. A cerebral biops y was performed to confirm brain involvement. Further analysis revealed the following marker profile: BCL-2/MYC^+^, CD30^–^ p53^+^, BCL-6^+^, BCL-2^–^, and MYC^–^. He died 1 year later, with no evidence of ocular recurrence.

**TABLE 1:** Variation in vitreous immune mediator values during IVT MTX/dexamethasone therapy from baseline to day 90.

Immune mediators	Day of therapy								
	Baseline	11	17	22	27	34	48	62	90
Chemokines									
CCL1	9	90	40	18	10	9	3	<3	<3
CCL2 (MCP-1)	5,547	3,929	3,168	3,058	2,732	3,272	4,093	4,200	3,748
CCL3 (MIP-1α)	11	10	6	3	<2	<2	<2	<2	<2
CCL4 (MIP-1β)	40	50	24	20	13	<9	27	22	16
CCL5 (RANTES)	6	11	7	5	4	2	6	5	3
CCL8 (MCP-2)	20	18	6	3	2	<1	<1	<1	<1
CCL11	<4	7	6	5	<4	<4	5	<4	<4
CCL17	1	1	1	1	<1	<1	<1	<1	<1
CCL19 (MIP-3β)	969	1,128	560	466	213	141	40	20	<12
CCL20 (MIP-3α)	3	16	27	25	27	20	<3	<3	<3
CCL23 (MIP-3)	144	303	204	144	120	102	49	46	<23
CXCL1	5	13	5	5	<3	<3	5	4	<3
CXCL8 (IL-8)	26	105	76	93	67	28	54	41	22
CXCL9 (MIG)	169	66	31	11	<5	<5	6	<5	19
CXCL10 (IP-10)	967	673	238	151	93	54	114	94	55
CXCL12 (SDF-lα)	611	943	937	832	661	498	298	230	119
CXCL13 (BLC)	4,351	3,209	1,112	705	382	258	96	63	<22
TNF-β	<12	<12	<12	<12	<12	<12	<12	<12	<12
Growth factors									
NGF-β	<8	<8	<8	<8	<8	<8	<8	<8	<8
BDNF	<3	<3	<3	<3	<3	<3	<	<3	<3
EGF	<3	6	<3	3	<3	<3	<	<3	<3
FGF-2	<4	<4	<4	<4	<4	<4	<4	<4	<4
HGF	1,582	2,910	2,447	2,468	1,914	2,087	1,964	1,872	1,574
LIF	53	108	43	43	42	62	57	44	40
PDGF-BB	<8	<8	<8	<8	<8	<8	<8	<8	<8
PIGF-1	<2	<2	<2	<2	<2	<2	<2	<2	<2
SCF	<2	3	4	4	<2	3	5	4	4
VEGF-A	40	39	21	21	20	26	29	23	16
BAFF	18	139	77	35	23	19	<8	<8	<8
GM-CSF	<18	<18	<18	<18	<18	<18	<18	<18	<18
G-CSF (CSF-3)	105	283	156	148	138	129	<22	<22	<22
Other soluble factors									
MMP-1	13	72	34	24	19	<13	14	<13	<13
Cytokines									
IL-lα	<1	<1	<1	<1	<1	<1	<1	<1	<1
IL-1RA	525	1,637	551	213	<54	<54	107	57	84
IL-1β	<3	<3	<3	<3	<3	<3	<3	<3	<3
IL-2	<8	<8	<8	<8	<8	<8	<8	<8	<8
IL-4	<13	<13	<13	<13	<13	<13	<13	<13	<13
IL-5	<13	<13	<13	<13	<13	<13	<13	<13	<13
IL-6	74.9	5000.0	5000.0	1428.0	1302.0	NA	95.1	50.9	40.5
IL-7	2	3	5	6	6	6	6	6	6
IL-9	<9	<9	<9	<9	<9	<9	<9	<9	<9
IL-10	4,619	2,729	56	<3	<3	<3	<3	<3	<3
IL-12p70	<13	<13	<13	<13	<13	<13	<13	<13	<13
IL-13	<6	<6	<6	<6	<6	<6	<6	<6	<6
IL-15	<4	<4	<4	<4	<4	<4	<4	<4	<4
IL-16	435	295	168	120	89	54	<12	<12	<12
IL-17A	4	<3	<3	<3	<3	<3	<3	<3	<3
IL-18	<18	<18	<18	<18	<18	<18	<18	<18	<18
IL-21	<13	<13	<13	<13	<13	<13	<13	<13	<13
IL-22	102	73	<26	<26	<26	<26	<26	<26	<26
IL-23	<19	<19	<19	<19	<19	<19	<19	<19	<19
IL-27	<26	<26	<26	<26	<26	<26	<26	<26	<26
IL-29 (IFN-A1)	121	484	338	223	144	71	<45	<45	<45
IL-31	<15	<15	<15	<15	<15	<15	<15	<15	<15
IFN-α2	<1	<1	<1	<1	<1	<1	<1	<1	<1
IFN-γ	<16	<16	<16	<16	<16	<16	<16	<16	<16
TNF-α	<12	24	22	<12	<12	<12	<12	<12	<12
IL-2R (SCD25)	1,504	1,330	843	333	192	<96	<96	<96	<96

NA, insufficient material.

## Methods

At each IVT chemotherapy session, following examination and imaging of the affected eye, topical anesthetics were repeatedly administered for adequate numbness. A valve 27G trocar was placed in the pars plana after topical disinfection with chlorhexidine. A 0.2 mL sample of vitreous fluid was removed from the eye using a tuberculin syringe fitted with a 30G needle. The eye was immediately injected with 0.1 mL of dexamethasone and 0.1 mL of the MTX preparation.

The vitreous sample was divided into two 0.1 mL aliquots and immediately stored at −15 °C until shipment to the laboratory for analysis within one week. While storage at −80 °C is considered optimal for cytokine preservation, the short interval between collection and analysis was expected to limit degradation. Samples were analyzed using a multiplex bead-based immunoassay platform for the simultaneous quantification of cytokines, chemokines and growth factors.

## Results

Nine serial samples were collected over 12 weeks. Fifty-eight soluble immune mediators were analyzed. The results are presented in Table 1.

**FIGURE 2:**
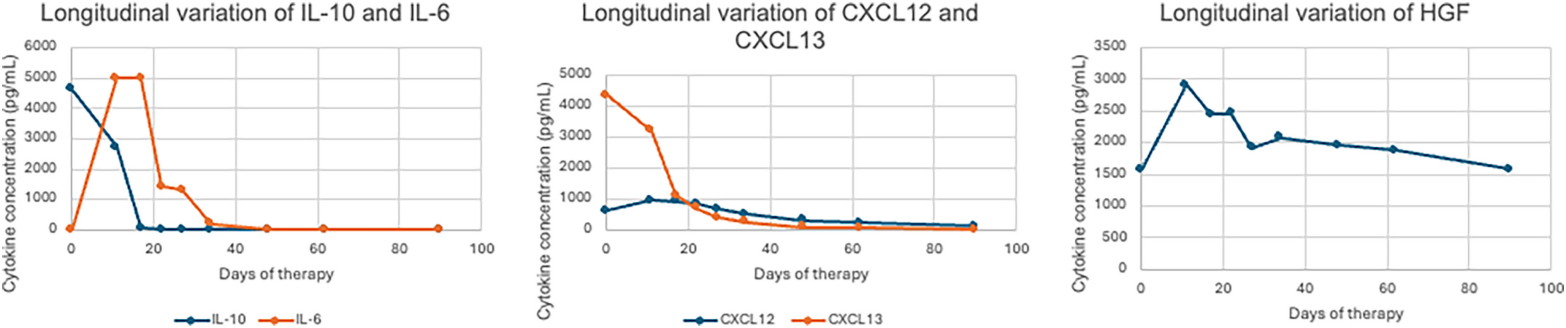
Longitudinal variation of selected vitreous immune mediators (pg/mL) during intravitreal methotrexate and dexamethasone therapy. Key cytokines (IL-10, IL-6), chemokines (CXCL12, CXCL13), and growth factor (HGF) are shown, illustrating differential kinetics during treatment. Line graph showing longitudinal changes in vitreous concentrations of multiple cytokines, chemokines, and growth factors during intravitreal methotrexate and dexamethasone treatment.

Among the cytokines, the following were found to vary during the course of follow-up: IL-1RA, IL-6, IL-29, IL-2R, and IL-10, which were initially elevated, decreased, and became undetectable by day 22. IL-16, which was elevated at baseline, exhibited a progressive decrease during treatment and became undetectable by day 48. The chemokines showing the greatest variations were MCP-1 (always elevated), CCL20, CCL23, IP-10, CXCL12, and CXCL13. Among the growth factors, HGF was constitutively elevated in all samples, ranging from 1,582 to 2,468 pg/mL on day 22 of therapy (Figure 2).

**FIGURE 3:**
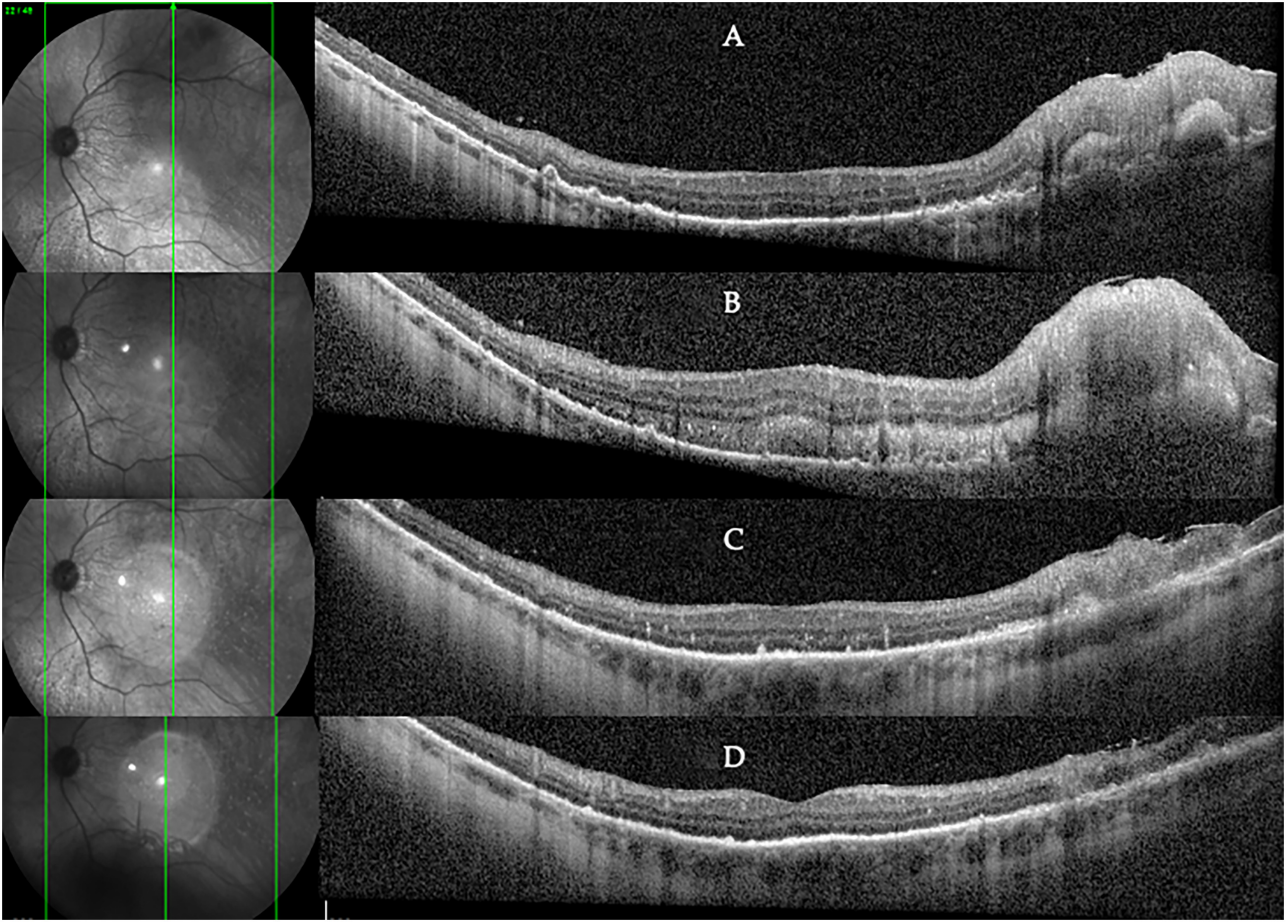
Optical coherence tomography (OCT) images showing subretinal hyperreflective infiltrates at baseline **(A)**, day 11 **(B)**, day 27 **(C)**, and day 48 **(D)**, with progressive regression and resolution **(D)** of the lesion following intravitreal MTX/dexamethasone therapy.

Anatomically, the ocular tumor appeared to regress by day 48 (Figure 3). The therapy was continued for an additional 52 days to achieve consolidation. The treatment was interrupted upon the initiation of systemic chemotherapy. He passed away a year after the last intravitreal dose due to complications of systemic chemotherapy, but no ocular recurrence had developed.

## Discussion

Our study documented the variation in soluble mediators over 90 days in a single patient with PVRL, during which the patient received IVT chemotherapy consisting of MTX and dexamethasone. The widely differing behaviors observed among various cytokines may help characterize the dynamic interplay between the tumor and host throughout the treatment period.

Usui et al. measured immune mediators in patients with VRL and uveitis. They found that vitreous levels of IL-10, CXCL13, bFGF, MCP-1, MIP-1α, MIP-1β, SDF-1α, and VEGF were significantly higher in patients with VRL than in those with uveitis (11). In our patient, we found elevations within the same range, except for bFGF, which was not elevated. In addition, certain cytokines that were not previously tested showed elevated levels at baseline. Among the cytokines that showed significant variations during therapy, we identified the following as reaching low or undetectable levels during therapy: MIP-1α, MIP-1β, MCP-2, MIP-3β (CCL19), MIP-3 (CCL23), MIG (CXCL9), IP-10 (CXCL10), SDF-1α (CXCL12), BLC (CXCL13), VEGFα, CSF-3, IL-1RA, IL-10, IL-16, IL-22, IFN-A1 (IL-29), and IL-2R. Some cytokines remained above the detection level: MCP-1 (CCL2), IL-8, HGF, LIF, and IL-6.

IL-10 is a pleiotropic cytokine produced by B-cells, T cells, and monocytes. It acts as a growth factor for both normal and lymphomatous human B-cells, where it has an autocrine function. IL-10 is secreted by over 90% of VRL and is a key biomarker in the diagnosis of VRL, whereas interleukin-6 (IL-6) is more commonly associated with inflammation. IL-10 protects the tumor from a cytotoxic response by inhibiting cytokine release from activated T and NK cells and blocking the antigen-presenting activity of macrophages (1, 3, 5). Monitoring IL-10 levels during therapy provides valuable insights into the treatment response. Gu et al. reported that IL-10 could also serve as a marker of recurrence when IL-10 levels reach 50 pg/mL or more, after an initial decline (5). In our case, IL-10 levels were initially elevated and then declined rapidly during treatment, consistent with previous findings (5). IL-6 is produced by several inflammatory cell types. It serves as an auxiliary marker for VRL diagnosis (12). It often becomes transiently elevated following the initiation of IVT chemotherapy and probably reflects the reactivation of the immune response against the lymphoma. It is also elevated in cases of infection following intravitreal injections and is also observed in systemic non-Hodgkin’s lymphoma (NHL) (12, 13).

Interleukin-1 receptor antagonist (IL-1RA) and soluble interleukin-2 receptor (sIL-2R/sCD25) are markers of host immune response against malignancies (14, 15). While IL-1 is required for lymphocyte activation, IL-1RA, by binding soluble IL-1, lowers its ability to respond to the tumor (14). In DLBCL, serial measurements of sIL-2R in peripheral blood during chemotherapy have prognostic value: an early decline in sIL-2R levels is associated with improved response and remission, whereas persistently elevated levels suggest a poorer prognosis (15). In our case, the transient elevation of IL-1RA, soluble IL-2R, and IL-6 during therapy likely reflected the activation of host immune responses. This interpretation, however, should be considered in light of the potential confounding effect of concomitant dexamethasone administration. Dexamethasone is a potent anti-inflammatory agent that may contribute to the reduction of cytokine levels independently of tumor regression. Therefore, the observed decline in inflammatory mediators such as IL-10, IL-6, IL-16, IL-1RA, and sIL-2R likely reflects a combined effect of both cytotoxic tumor reduction induced by methotrexate and non-specific immunosuppressive effects of dexamethasone. However, the differential kinetics observed among mediators, particularly the slower decline of CXCL12 and CXCL13 compared with IL-10, suggests that these changes may not be solely attributable to corticosteroid effects but rather reflect persistent tumor–microenvironment interactions.

CXCL13 (BLC) plays a pivotal role in B-cell differentiation, antigen interaction, and homing. In PCNSL, both CXCL13 and its receptor CXCR5 are expressed by malignant B-cells and the vascular endothelium but are absent in normal CNS tissue (16). In PVRL, CXCL13 is expressed at the level of the retinal pigment epithelium (RPE), together with SDF-1 (CXCL12), whereas CXCR5 and CXCR4 (the receptor for CXCL12) are detected on lymphomatous B-cells. This pattern supports the hypothesis that both CXCL12 and CXCL13 facilitate the migration of malignant cells from the choroidal circulation to the subretinal space, contributing to intraocular infiltration (17). Similarly, high levels of CXCL12 are expressed in the brain tissue of patients with PCNSL (18). The initial elevated levels of CXCL12 and CXCL13 in our patient were consistent with the findings of Usui et al. (11). Following IVT therapy, the decline in both chemokines proceeded at a slower pace than that of IL-10, the latter being a good marker of the persistence of lymphoma cells in the eye. Persistence of either or both chemokines produced by endothelial and RPE cells could further facilitate resurgence of lymphomatous cells into the subretinal space and vitreous, or be a marker of poor therapeutic response. Fisher et al. reported reductions in CSF levels of CXCL12 and CSCL13 in PCNSL patients responding to chemotherapy, while rising levels in those that did not (19). Currently, there are no clear guidelines regarding when intraocular chemotherapy can be safely discontinued. Reaching undetectable CXCL12/CXCL13 levels may be a valid therapeutic target, at which point, if the absence was sustained, further chemotherapy could be discontinued. However, this needs to be confirmed in longitudinal studies.

MCP-1 primarily functions as a chemoattractant for circulating monocytes. Upon differentiation into macrophages, MCP-1 binding to CCR2 decreases, resulting in a diminished cellular response to MCP-1. Monocytes recruited from the systemic circulation tend to be tumor suppressants with an M1 profile. Tumor-infiltrating monocytes, which also proliferate under the influence of MCP-1 (driven by NF-κB), have an M2 immunosuppressive profile (20). Without knowing the source of infiltrating macrophages, it is difficult to establish the exact role of MCP-1 in the survival or destruction of PVRL cells.

We observed moderately elevated levels of vitreous MIP-1α and MIP-1β in our patient, which progressively decreased during IVT therapy. Both MIP-1α and MIP-1β are secreted by normal and malignant B-cells in response to BCR stimulation. They serve to recruit activated CD4+ and CD8+ T cells, monocytes, NK cells, and other inflammatory cells. In DLBCL, high serum concentrations (4–5 times higher than those observed in the vitreous) are associated with a poor survival interval but also indicate susceptibility to certain Burton kinase inhibitors (21). However, its role in PVL requires further elucidation.

The activation of the NF-κB pathway is a well-established mechanism by which tumor cells evade apoptosis. Mutations in MYD88 constitutively activate NF-κB and, as discussed above, significantly increase the production of MCP-1. Similarly, FGF2 is upregulated by NF-κB, as seen in several PVL cases reported by Usui et al. but not in our patient (11). While FGF2 is a key tumor-promoting factor in the growth and metastasis of many solid tumors, its role in hematologic tumors is less well-defined (22). In NHL, serum levels were not associated with disease stage but rather with tumor bulkiness. Our patient’s tumor was largely limited to the subretinal space of the involved eye, whereas in most of Usui’s patients, the involvement was more diffuse in both the vitreous and subretinal spaces. FGF2 may be a marker of tumor bulk and, as such, may serve the same purpose in CNS LBCL.

Cytokine-stimulated RPE cells have been shown to produce chemokines Mig and IP-10. These cells also express TLRs (frequently coactivated with BCR signaling through mutations in MYD88) on their surface and can function as antigen-presenting cells (APCs) (7, 23). Mig and IP-10 share several biological features: both are induced by IFN-γ, bind to the CXCR3 receptor (expressed on lymphomatous B-cells in CNSL) and act as homing signals for activated lymphocytes. While Mig is believed to promote autocrine proliferation of B-cells, IP-10 levels in patients with uveitis are associated with T-cell infiltration into ocular tissues (10, 24). Furthermore, Usui et al. reported elevated IP-10 concentrations in cases of VRL with subretinal infiltration, a pattern often accompanied by reactive T cells in the vitreous (11). These findings suggest that IP-10 and Mig are components of the local host immune response to lymphomatous infiltration.

In our patient, longitudinal analysis showed a marked decrease in intraocular Mig and IP-10 levels following IVT MTX/dexamethasone therapy. Their initial upregulation, followed by a progressive decline, likely reflects an active host tissue response to both lymphoma and therapy, underscoring the potential role of these chemokines as biomarkers of disease activity and as tools for treatment monitoring in VRL.

Hepatocyte growth factor (HGF) is secreted by mesenchymal cells and functions as a multifunctional cytokine, primarily acting on epithelial-derived cells by binding to the proto-oncogenic c-MET receptor. The HGF/c-MET signaling axis has been investigated as a therapeutic target for various malignancies. In B-cell lymphomas, HGF increases the adhesion of c-MET–positive cells to extracellular matrix components, such as fibronectin and collagen, and promotes the migration and invasion of lymphoma cells. These findings may explain the association between DLBCLs expressing HGF and/or c-MET and poor prognosis (25).

In our patient, HGF levels were markedly elevated both at baseline and throughout IVT therapy. Notably, despite initial ocular tumor regression, the patient developed central nervous system involvement at week 12, required systemic chemotherapy, and died 1 year later. The persistently elevated intraocular HGF levels observed in this case may therefore suggest an association with aggressive disease, consistent with prior literature. Further studies are needed to clarify the role of the HGF/c-MET pathway in VRL.

The findings of our study illustrate the complexity of the interactions between the tumor and the immune microenvironment in a patient with PVRL and suggest that dynamic changes in vitreous cytokine levels may serve as informative biomarkers for disease activity, therapeutic response, tumor bulk, and possibly prognosis. Not all cytokines identified by Usui et al. were elevated in our patient, including some with a clear role in the immune response against the tumor, such as IFN-γ, TNF-α, and IL-17, indicating that the individual patient response is variable. Prospective follow-up of a number of patients receiving therapy would help elucidate the role of each cytokine/chemokine. In addition, it can serve as a basis to determine the length of therapy required if one uses the criterion that the implicated cytokines need to reach an undetectable level.

## Data Availability

The original contributions presented in this study are included in this article/supplementary material, further inquiries can be directed to the corresponding author.
